# Artificial intelligence in respiratory care: knowledge, perceptions, and practices—a cross-sectional study

**DOI:** 10.3389/frai.2024.1451963

**Published:** 2024-09-03

**Authors:** Jithin K. Sreedharan, Asma Alharbi, Amal Alsomali, Gokul Krishna Gopalakrishnan, Abdullah Almojaibel, Rawan Alajmi, Ibrahim Albalawi, Musallam Alnasser, Meshal Alenezi, Abdullah Alqahtani, Mohammed Alahmari, Eidan Alzahrani, Manjush Karthika

**Affiliations:** ^1^Department of Respiratory Therapy, College of Health Sciences, University of Doha for Science and Technology, Doha, Qatar; ^2^Department of Respiratory Care, Prince Sultan Military College of Health Sciences, Dammam, Saudi Arabia; ^3^Department of Respiratory Care, Batterjee Medical College, Jeddah, Saudi Arabia; ^4^Department of Respiratory Care, Imam Abdulrahman Bin Faisal University, Dammam, Saudi Arabia; ^5^Advanced Center for Clinical Simulation, Prince Sultan Military College of Health Sciences, Dammam, Saudi Arabia; ^6^Dammam Health Network, Eastern Health Cluster, Dammam, Saudi Arabia; ^7^Department of Physical Therapy, Prince Sultan Military College of Health Sciences, Dammam, Saudi Arabia; ^8^Department of Health and Medical Sciences, Liwa College, Abu Dhabi, United Arab Emirates

**Keywords:** artificial intelligence, AI, respiratory care, respiratory therapy, professionals, challenges, integration artificial intelligence, integration

## Abstract

**Background:**

Artificial intelligence (AI) is reforming healthcare, particularly in respiratory medicine and critical care, by utilizing big and synthetic data to improve diagnostic accuracy and therapeutic benefits. This survey aimed to evaluate the knowledge, perceptions, and practices of respiratory therapists (RTs) regarding AI to effectively incorporate these technologies into the clinical practice.

**Methods:**

The study approved by the institutional review board, aimed at the RTs working in the Kingdom of Saudi Arabia. The validated questionnaire collected reflective insights from 448 RTs in Saudi Arabia. Descriptive statistics, thematic analysis, Fisher’s exact test, and chi-square test were used to evaluate the significance of the data.

**Results:**

The survey revealed a nearly equal distribution of genders (51% female, 49% male). Most respondents were in the 20–25 age group (54%), held bachelor’s degrees (69%), and had 0–5 years of experience (73%). While 28% had some knowledge of AI, only 8.5% had practical experience. Significant gender disparities in AI knowledge were noted (*p* < 0.001). Key findings included 59% advocating for basics of AI in the curriculum, 51% believing AI would play a vital role in respiratory care, and 41% calling for specialized AI personnel. Major challenges identified included knowledge deficiencies (23%), skill enhancement (23%), and limited access to training (17%).

**Conclusion:**

In conclusion, this study highlights differences in the levels of knowledge and perceptions regarding AI among respiratory care professionals, underlining its recognized significance and futuristic awareness in the field. Tailored education and strategic planning are crucial for enhancing the quality of respiratory care, with the integration of AI. Addressing these gaps is essential for utilizing the full potential of AI in advancing respiratory care practices.

## Introduction

The digitalisation of medical record has resulted in the accumulation of a huge amount of data (Salmon et al., 2021). IBM estimates that approximately one million gigabytes of data over the lifetime of an average patient and with the doubling of healthcare data every few years (IBM) (Carson, 2015). It is crucial to harness the power of artificial intelligence (AI) and computer scientists to make sense of this big data (Nagendran et al., 2020). AI is revolutionizing various industries, including healthcare, with applications like machine learning algorithms transforming medical diagnosis, treatment planning, and patient monitoring (Maleki Varnosfaderani and Forouzanfar, 2024; Alowais et al., 2023; Harry, 2023). In respiratory care, AI has shown promise in interpreting lung function tests, analyzing respiratory sounds, and managing ventilated patients (Al-Anazi et al., 2024; Stivi et al., 2024).

As AI technologies advance, it is essential to consider the viewpoints of healthcare professionals who will be impacted by the use of these tools (Yadav et al., 2024). Respiratory therapists, in particular, are crucial in diagnosing and treating respiratory conditions. Their opinions and perspectives regarding AI in their field can impact how these technologies are adopted and utilized effectively (Ahuja and Nair, 2021; Honkoop et al., 2022; Sreedharan and Varghese, 2020).

Previous research has examined healthcare professionals’ attitudes and perceptions towards AI in various medical specialties, including radiology (Allam et al., 2023), pathology (Berbís et al., 2023), and surgery (Li et al., 2022). However, there is a paucity of research specifically focused on respiratory care professionals (Honkoop et al., 2022). It is crucial to understand their perspectives to improve AI literacy, overcome potential obstacles, and promote the effective implementation of AI tools and algorithms in respiratory care. This study sought to evaluate the knowledge, perception, and practices of respiratory care professionals from the Kingdom of Saudi Arabia, on AI tools and related applications.

## Methods

### Study design and participants

This was a cross-sectional survey study conducted online. The survey targeted respiratory therapists and educators across various institutions in Saudi Arabia, Participants were recruited via email invitation and notification, which contained a link to the web version of the questionnaire. The professional society for RTs in the country, along with leaders of the profession, disseminated the online questionnaire among their members and networks. The online version of the questionnaire was set up using the SurveyMonkey^®^ platform (SurveyMonkey Inc., Palo Alto, CA) (Abd Halim et al., 2018). The software was designed to be completely anonymous, and it did not collect respondents’ IP addresses. Participation in the survey was voluntary, and all participants were instructed not to fill out the survey more than once. Weekly reminders were sent to maximize participation rates. The target sample size, determined using the appropriate formula, was 560 (with a range of 535–585). The survey was conducted from *February 2023 to July 2023*, resulting in a sample of 448 respondents. This sample size was calculated with an assumed response distribution of 50%, a margin of error of ±5%, and a confidence level of 95%.

### Survey instrument

The survey questionnaire was developed in English. Relevant literature on applications of artificial intelligence (AI) in respiratory therapy and the competencies required for its uses were reviewed. Based on this, two researchers with expertise in medical education, AI in respiratory care, and survey design constructed the questionnaire to assess RTs knowledge and perceptions about AI. Content validation was performed by a five-member expert panel comprising the research group with proficiency in survey analysis and English language. The panel evaluated the core content, language appropriateness, question suitability across domains, scoring patterns, etc. The survey was piloted with an experimental group of 20 judiciously selected participants varying in age, gender, and educational backgrounds. Questions in the knowledge, perception and practice domains underwent content validity and internal consistency testing, with responses analyzed using Cronbach’s alpha (overall *α* = 0.91, acceptable). The expert panel reviewed observations and recommended modifications before survey launch.

The questionnaire had four sections: socio-demographics (part 1), knowledge (part 2), perception (part 3), and practice (part 4). Socio-demographics collected were gender, age, nationality, location, education, designation, experience, hospital type/bed number, and specialization area. Participants rated importance of AI in respiratory care on a Likert scale with the response options ratings from “Strongly agree” to “Strongly disagree” options, with one 1–5 rating scale question (Jebb et al., 2021). All responses were anonymized and data was securely collected in a password-protected database.

### Data analysis

Descriptive statistics were calculated for quantitative survey responses [frequencies and (percentages), or means, ±standard deviations as appropriate]. Responses to open-questions were analyzed thematically. The chi-square test or Fisher’s exact test were used to assess differences in responses between genders and years of experience. A *p*-value <0.05 was considered statistically significant. All data analyses were conducted using R statistical software version 4.0.2.

### Ethical considerations

The survey was approved by the Institutional Review Board of Prince Sultan Military College of Health Sciences in Dhahran, Saudi Arabia (IRB number: IRB-2022-RC-008). Participants provided informed consent by clicking the survey link, which displayed the consent information before the questionnaire. Those who did not want to participate were given the option to decline. The consent informed participants of the study’s objectives and ensured the anonymity of the survey, protecting their data privacy. This study followed the guidelines set out in the Declaration of Helsinki.

## Results

The survey included 230 (51%) females and 218 (49%) males. The majority (54%) were in the 20–25-year age group, and most participants (69%) held a Bachelor’s degree in respiratory care, The majority (77%) graduated from universities in Saudi Arabia, approximately 52% were currently working in hospitals and 73% of them had 0–5 years of experience (Table 1).

**Table 1:** Demographic characteristics of the study participants.

Characteristic	Overall, *N* = 448
Age (in years)	
20–25	241 (54%)
26–30	90 (20%)
31–35	57 (13%)
Above 35	48 (11%)
Below 20	12 (2.7%)
Highest education in RT	
Bachelor degree	311 (69%)
Diploma	38 (8.5%)
Doctoral degree	26 (5.8%)
Master degree	73 (16%)
Country of graduation	
India	16 (3.6%)
Other Arab universities	24 (5.4%)
Philippines	9 (2.0%)
Saudi Arabia	343 (77%)
Western education	56 (12%)
Working presently in	
A hospital	230 (52%)
An academic institution	148 (34%)
Government	2 (0.5%)
Students (masters)	61 (14%)
Not working	7 (1.56%)
Experience (in years)	
0 to 5	327 (73%)
6 to 10	59 (13%)
10 to 20	46 (10%)
Above 20	16 (3.6%)

### AI knowledge and perceptions

The survey revealed varied levels of familiarity with AI among respiratory care professionals. About 28% of respondents were comfortable with the concept of AI but lacked technical knowledge, while 27% were familiar with AI but not confident in applying it to their work. Another 20% had only heard of AI through the news or media, 16% had no understanding of AI, and 8.5% had practical experience working with AI. Despite the varying levels of familiarity, there was strong support for integrating AI into respiratory therapy education. A majority, 59%, agreed or strongly agreed that basic AI knowledge should be included in the curriculum. When examining the sources of AI knowledge, 34% of participants were self-taught, 20% gained knowledge through courses, 20% through work-related activities, while 12% acquired it via postgraduate training, and 14% had no AI knowledge at all.

Looking to the future, 51% of respondents believed that AI would play a significant role in respiratory care, and 55% anticipated that AI would be integrated into many aspects of respiratory care practices. However, perspectives on AI’s impact were mixed, with 29% remaining neutral, 27% disagreeing, and only 18% expressing concern that AI might disrupt or threaten respiratory care practice. Similarly, opinions on AI’s limitations were divided, with 33% neutral, and 20% each agreeing or disagreeing about the absence of AI limitations in their work. Organizational readiness for AI implementation varied widely. While 18% of respondents were in charge of AI at their institutions, 82% were not. Among the respondents, 41% reported having someone responsible for AI in education, research, innovation, and integration within their organization. However, 39% were unaware of their organization’s AI strategy, 30% confirmed that their organization lacked one, 16% noted that a strategy was in development, and 15% reported having an established AI strategy.

The key challenges identified for AI implementation were primarily knowledge gaps and skill development, each cited by 23% of respondents. Other challenges included the lack of AI training in universities (17%), difficulty in finding quality AI education/training courses (15%), and the challenges of integrating AI into work practices (11%) and training current staff (11%). Participants highlighted several AI applications as crucial for respiratory care, including research (19%), critical care management (18%), image interpretation (17%), and quality control (15%). Other areas of interest were dose management (7.8%), communication (7.6%), emergency patient care (5.4%), teaching and mentoring (9.2%), and policy/procedures development (0.2%).

### Based on the gender

This study reveals significant gender differences in AI knowledge and perceptions among respiratory care professionals (Table 2). Female respondents were predominantly younger, with 66% aged 20–25 compared to 41% of males. However, more males held doctoral degrees (9.6% vs. 2.2% of females) and were graduates of Western universities (17% vs. 8.7% of females). These differences indicate a gender gap in certain areas of AI exposure and education, although both groups generally recognized the importance of AI in respiratory care.

**Table 2:** Knowledge, perception, and practice about AI in RC domain among the participants based on gender.

Characteristic	Overall, *N* = 448	Female, *N* = 230	Male, *N* = 218	*p*-value
How well you understood what AI means				
I have no idea	73 (16%)	48 (21%)	25 (11%)	
I’m comfortable with what it means, but I’ve no technical knowledge	124 (28%)	50 (22%)	74 (34%)	
I’m familiar with it but would not confidently apply that knowledge at work	123 (27%)	83 (36%)	40 (18%)	<0.001
I’ve read/heard about it in the news, poster or social media	90 (20%)	42 (18%)	48 (22%)	
Very comfortable, I work in AI	38 (8.5%)	7 (3.0%)	31 (14%)	
The RT curriculum should include at least some basic knowledge of AI				
Agree	154 (34%)	84 (37%)	70 (32%)	
Disagree	24 (5.4%)	10 (4.3%)	14 (6.4%)	
Neutral	90 (20%)	45 (20%)	45 (21%)	0.7
Strongly agree	110 (25%)	53 (23%)	57 (26%)	
Strongly disagree	70 (16%)	38 (17%)	32 (15%)	
What is your perception toward integrating AI into RC practice				
Aware of the challenge	110 (25%)	63 (27%)	47 (22%)	
Excited	117 (26%)	44 (19%)	73 (33%)	
I do not know enough	83 (19%)	54 (23%)	29 (13%)	<0.001
Neutral	97 (22%)	57 (25%)	40 (18%)	
Worried about the impact	41 (9.2%)	12 (5.2%)	29 (13%)	
Knowledge gain				
Attending courses	89 (20%)	55 (24%)	34 (16%)	
No knowledge	63 (14%)	34 (15%)	29 (13%)	
Postgraduate training	54 (12%)	28 (12%)	26 (12%)	0.2
Self-taught	151 (34%)	69 (30%)	82 (38%)	
Work-related activities	91 (20%)	44 (19%)	47 (22%)	
AI play an important role in RC				
Agree	121 (27%)	63 (27%)	58 (27%)	
Disagree	29 (6.5%)	10 (4.3%)	19 (8.7%)	
Neutral	129 (29%)	65 (28%)	64 (29%)	0.3
Strongly agree	108 (24%)	62 (27%)	46 (21%)	
Strongly disagree	61 (14%)	30 (13%)	31 (14%)	
AI will take place in many RC applications and practice				
Agree	153 (34%)	85 (37%)	68 (31%)	
Disagree	43 (9.6%)	15 (6.5%)	28 (13%)	
Neutral	94 (21%)	51 (22%)	43 (20%)	0.1
Strongly agree	96 (21%)	52 (23%)	44 (20%)	
Strongly disagree	62 (14%)	27 (12%)	35 (16%)	
AI will threaten/disrupt the respiratory care practice				
Agree	79 (18%)	41 (18%)	38 (17%)	
Disagree	119 (27%)	66 (29%)	53 (24%)	
Neutral	128 (29%)	71 (31%)	57 (26%)	0.11
Strongly agree	47 (10%)	24 (10%)	23 (11%)	
Strongly disagree	75 (17%)	28 (12%)	47 (22%)	
AI will threaten/disrupt some respiratory care careers				
Agree	96 (21%)	61 (27%)	35 (16%)	
Disagree	101 (23%)	52 (23%)	49 (22%)	
Neutral	123 (27%)	61 (27%)	62 (28%)	0.066
Strongly agree	57 (13%)	25 (11%)	32 (15%)	
Strongly disagree	71 (16%)	31 (13%)	40 (18%)	
AI has no limitations in my work				
Agree	90 (20%)	56 (24%)	34 (16%)	
Disagree	89 (20%)	37 (16%)	52 (24%)	
Neutral	149 (33%)	79 (34%)	70 (32%)	0.061
Strongly agree	45 (10%)	24 (10%)	21 (9.6%)	
Strongly disagree	75 (17%)	34 (15%)	41 (19%)	
Are you in charge of AI in your institution?				
No	367 (82%)	187 (81%)	180 (83%)	
Yes	81 (18%)	43 (19%)	38 (17%)	0.7
Someone responsible for AI in education research innovation and integration	184 (41%)	110 (48%)	74 (34%)	0.003
For my work AI is				
A big part of what we do	40 (8.9%)	18 (7.8%)	22 (10%)	
A small part	43 (9.6%)	21 (9.1%)	22 (10%)	0.009
I have no idea	107 (24%)	56 (24%)	51 (23%)	
It is future plan	137 (31%)	74 (32%)	63 (29%)	
We are not looking at or planning for	52 (12%)	16 (7.0%)	36 (17%)	
We’re beginner	69 (15%)	45 (20%)	24 (11%)	
Organization have a strategy for AI				
I have no idea	175 (39%)	89 (39%)	86 (39%)	
No	134 (30%)	60 (26%)	74 (34%)	
We’re developing one	72 (16%)	50 (22%)	22 (10%)	0.006
Yes	67 (15%)	31 (13%)	36 (17%)	
What are the biggest challenges for AI implementation in your work?				
Graduates aren’t leaning the AI knowledge and skills at university	76 (17%)	45 (20%)	31 (14%)	
It is hard to educate and train the current staff in AI technology	49 (11%)	23 (10%)	26 (12%)	
It is hard to find good education and training courses in AI	69 (15%)	34 (15%)	35 (16%)	
It is hard to implement AI in the work and practice	48 (11%)	17 (7.4%)	31 (14%)	0.2
Knowledge	101 (23%)	53 (23%)	48 (22%)	
The skill development	105 (23%)	58 (25%)	47 (22%)	
Which AI relating application do you most need in your work?				
All of the above	3 (0.7%)	2 (0.9%)	1 (0.5%)	
Communication	34 (7.6%)	17 (7.4%)	17 (7.8%)	
Critical care unit (managing critically ill)	81 (18%)	41 (18%)	40 (18%)	
Dose management (Ex. drug)	35 (7.8%)	13 (5.7%)	22 (10%)	
Emergency patient care	24 (5.4%)	18 (7.8%)	6 (2.8%)	
Image interpretation	75 (17%)	39 (17%)	36 (17%)	0.29
Policy and procedures	1 (0.2%)	0 (0%)	1 (0.5%)	
Quality control	68 (15%)	29 (13%)	39 (18%)	
Research in respiratory care	86 (19%)	52 (23%)	34 (16%)	
Teaching and mentoring (respiratory care education)	41 (9.2%)	19 (8.3%)	22 (10%)	

2 Pearson’s chi-squared tests; Fisher’s exact test.

**Figure 1:**
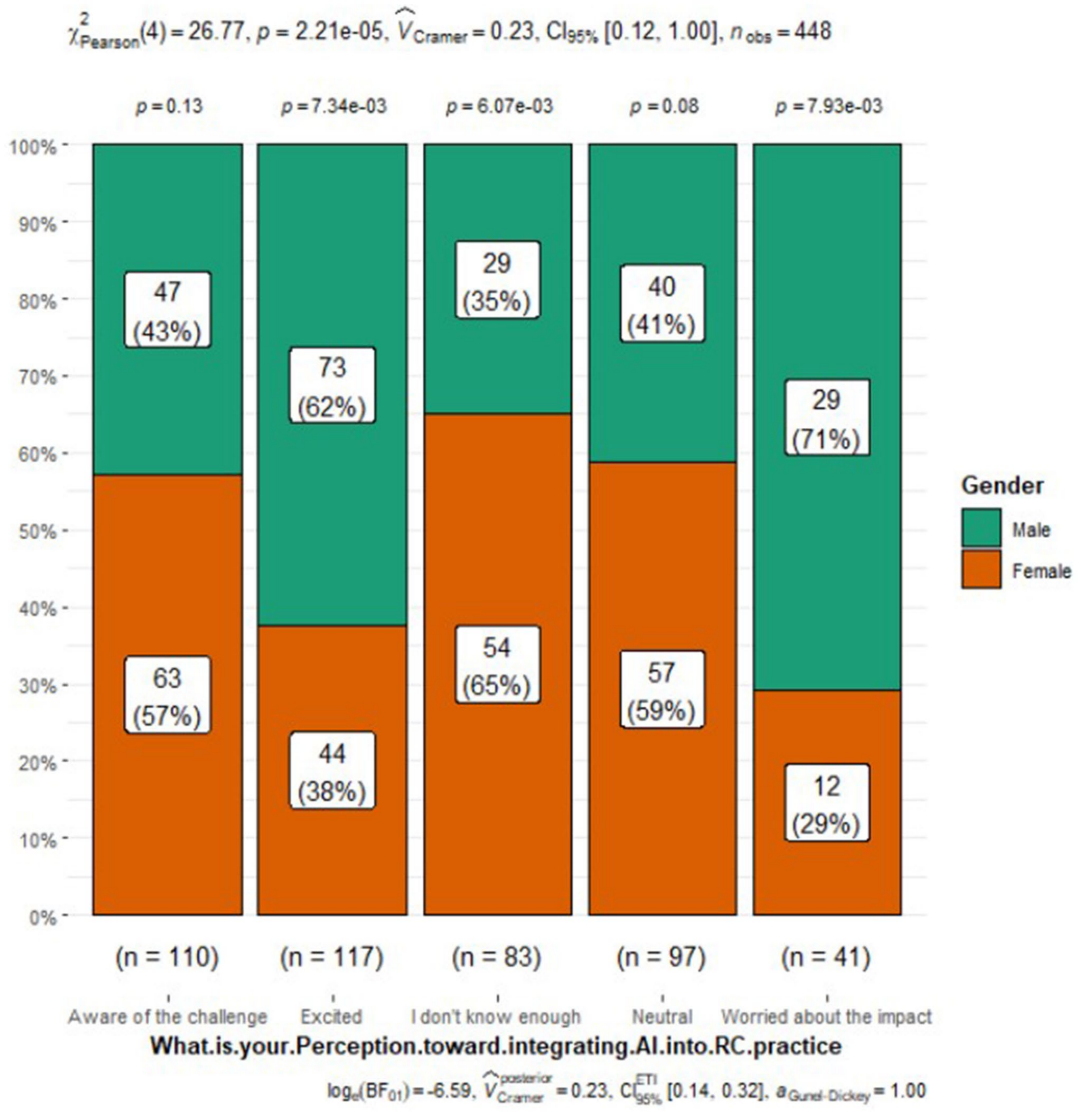
Perception toward integrating AI into RC practice.

**Figure 2:**
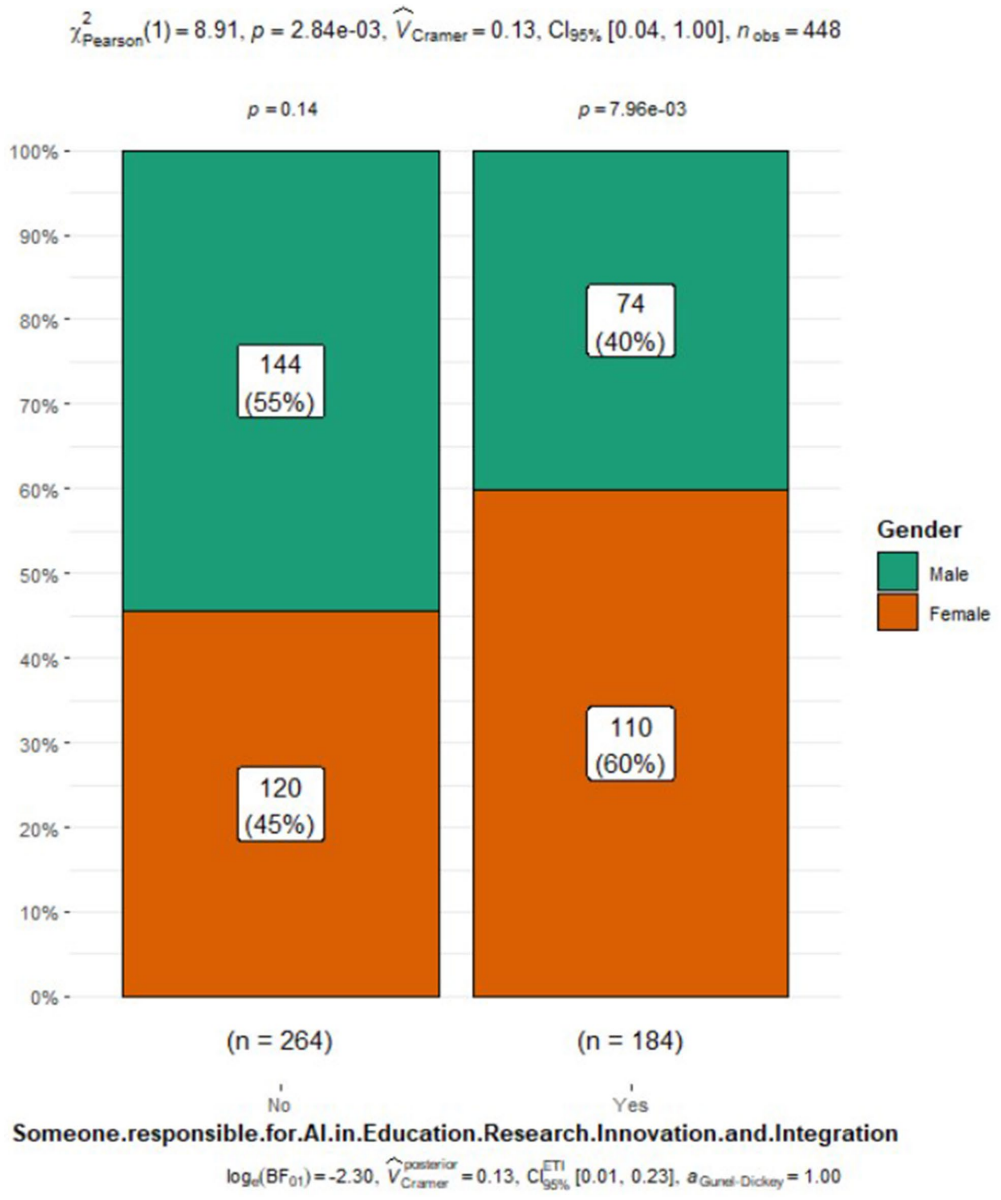
Responsible for AI in education, research, innovation, and research.

**Figure 3:**
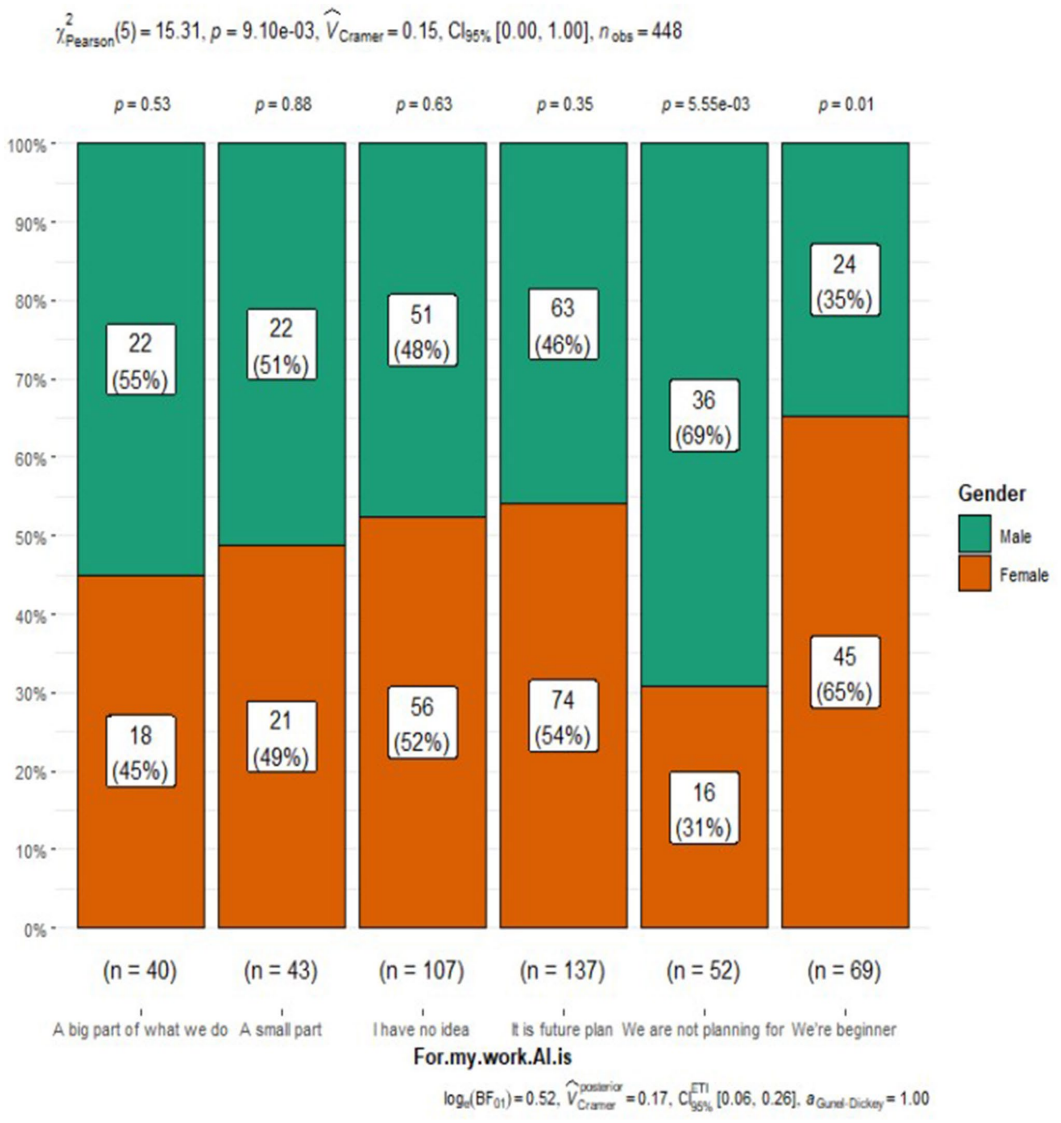
Role of AI in work.

**Figure 4:**
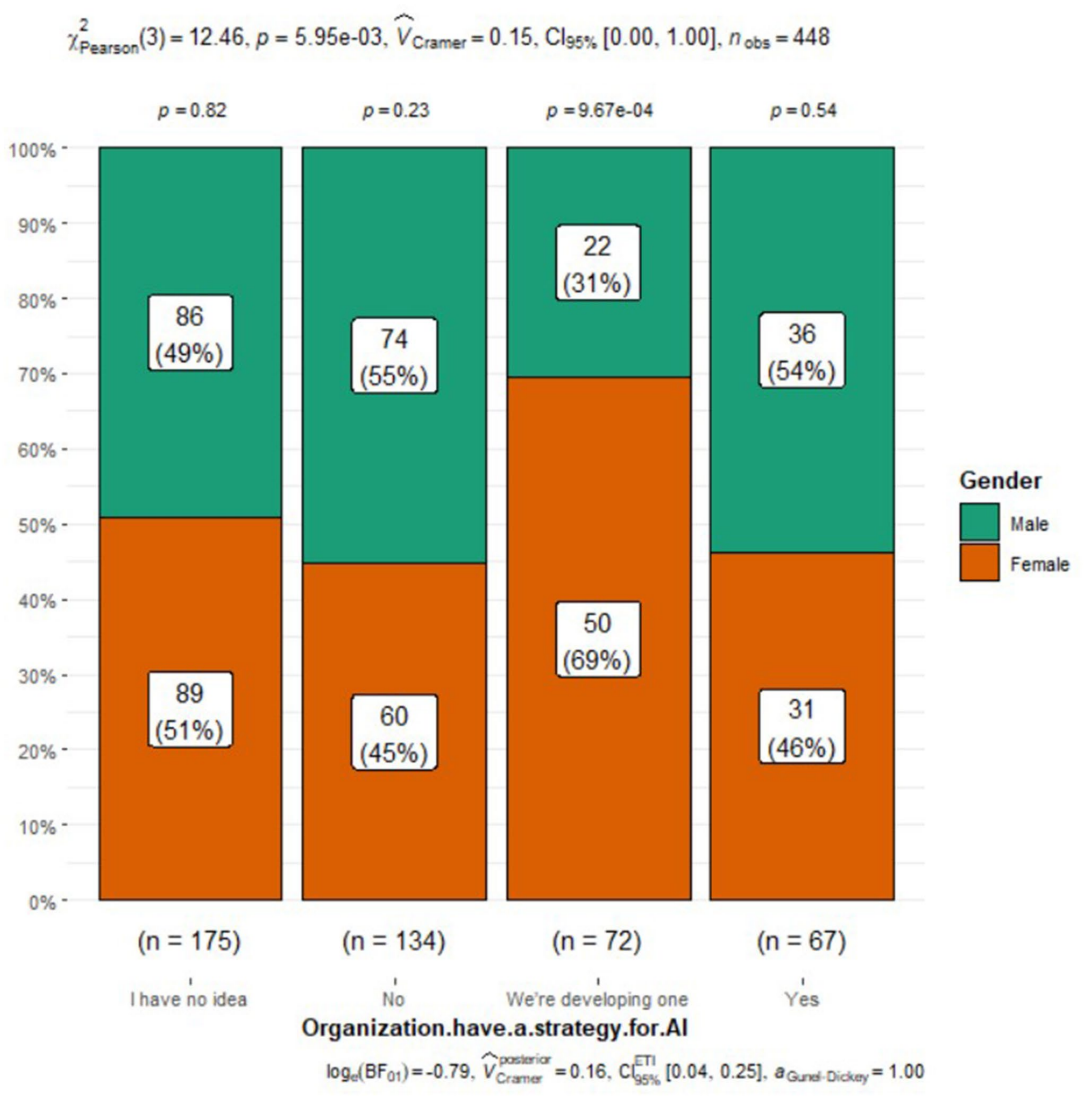
Organisation strategy for AI.

Perceptions of AI integration in respiratory care also varied by gender (Figure 1). Males were more likely to express excitement about AI (33% vs. 19% of females), while females were more inclined to acknowledge the challenges (27% vs. 22% of males) or admit to not knowing enough about AI (23% vs. 13% of males). Additionally, a slightly higher percentage of males believed that AI would be integrated into many respiratory care applications (37% vs. 31% of females). However, both genders had similar views on AI’s role, its potential threats to practice, and the need to include AI in the respiratory therapy curriculum. Interestingly, a higher proportion of females (48%) reported having someone responsible for AI education, research, or integration at their organization compared to males (34%) (Figure 2). Despite the differences in excitement levels, both male (46%) and female (41%) participants largely disagreed or strongly disagreed with the notion that AI would threaten or disrupt respiratory care practice in the near future, though a significant portion remained neutral (31% of females and 26% of males) (see Figures 3, 4).

In terms of AI familiarity, more males felt comfortable with the meaning of AI, and a greater percentage of males (14%) were currently working in AI-related fields compared to females (3%). Despite these differences, a majority of both male (58%) and female (60%) participants agreed or strongly agreed that the respiratory therapy curriculum should include basic AI knowledge.

### Based on the nature of RT professions

The survey included 448 respiratory care professionals, with 292 clinicians and 156 participants from academic institutions (Table 3). The findings revealed that a significant portion of respondents, recognized the potential of AI in respiratory care. Specifically, 34% overall, including 35% of RT clinicians, agreed that AI would play a crucial role in various respiratory care applications. However, awareness of organizational strategies for AI implementation was lacking, with 39% overall and 46% of RT clinicians admitting they had no idea about their organization’s AI plans. The survey also highlighted several key challenges in integrating AI into respiratory care practices. These included the insufficient AI knowledge and skills among graduates (17%), difficulties in training current staff (11%), and the scarcity of quality AI education courses (15%). These challenges underscore the need for targeted educational initiatives. When considering areas where AI could have the most impact, respondents identified critical care unit management (18%), research in respiratory care (19%), and image interpretation (17%) as the most crucial.

**Table 3:** Based on nature of RT professions about AI in RC domain among the participants.

Characteristic	Overall, *N* = 448	Clinician, *N* = 292	Academic institution, *N* = 156	*p*-value
How well you understood what AI means				
I have no idea	16 (3.6%)	10 (3.4%)	6 (3.8%)	<0.001
I’m comfortable with what it means, but I’ve no technical knowledge	46 (10%)	31 (11%)	15 (9.6%)	
I’m familiar with it but would not confidently apply that knowledge at work	73 (16%)	55 (19%)	18 (12%)	
I’ve read/heard about it in the news, poster or social media	124 (28%)	85 (29%)	39 (25%)	
Very comfortable, I work in AI	123 (27%)	55 (19%)	68 (44%)	
The RT curriculum should include at least some basic knowledge of AI				
Agree	90 (20%)	73 (25%)	17 (11%)	0.4
Disagree	38 (8.5%)	24 (8.2%)	14 (9.0%)	
Neutral	154 (34%)	101 (35%)	53 (34%)	
Strongly agree	24 (5.4%)	12 (4.1%)	12 (7.7%)	
Strongly disagree	90 (20%)	56 (19%)	34 (22%)	
What is your perception toward integrating AI into RC practice				
Aware of the challenge	110 (25%)	73 (25%)	37 (24%)	0.004
Excited	70 (16%)	50 (17%)	20 (13%)	
I do not know enough	110 (25%)	56 (19%)	54 (35%)	
Neutral	117 (26%)	83 (28%)	34 (22%)	
Worried about the impact	83 (19%)	61 (21%)	22 (14%)	
Knowledge gain				
Attending courses	97 (22%)	62 (21%)	35 (22%)	0.001
No knowledge	41 (9.2%)	30 (10%)	11 (7.1%)	
Postgraduate training	89 (20%)	47 (16%)	42 (27%)	
Self-taught	63 (14%)	54 (18%)	9 (5.8%)	
Work-related activities	54 (12%)	36 (12%)	18 (12%)	
AI play an important role in RC				
Agree	151 (34%)	95 (33%)	56 (36%)	0.062
Disagree	91 (20%)	60 (21%)	31 (20%)	
Neutral	121 (27%)	84 (29%)	37 (24%)	
Strongly agree	29 (6.5%)	17 (5.8%)	12 (7.7%)	
Strongly disagree	129 (29%)	92 (32%)	37 (24%)	
AI will take place in many RC applications and practice				
Agree	108 (24%)	59 (20%)	49 (31%)	>0.9
Disagree	61 (14%)	40 (14%)	21 (13%)	
Neutral	153 (34%)	101 (35%)	52 (33%)	
Strongly agree	43 (9.6%)	30 (10%)	13 (8.3%)	
Strongly disagree	94 (21%)	60 (21%)	34 (22%)	
AI will threaten/disrupt the respiratory care practice				
Agree	96 (21%)	60 (21%)	36 (23%)	0.8
Disagree	62 (14%)	41 (14%)	21 (13%)	
Neutral	79 (18%)	54 (18%)	25 (16%)	
Strongly agree	119 (27%)	74 (25%)	45 (29%)	
Strongly disagree	128 (29%)	86 (29%)	42 (27%)	
AI will threaten/disrupt some respiratory care careers				
Agree	47 (10%)	28 (9.6%)	19 (12%)	0.5
Disagree	75 (17%)	50 (17%)	25 (16%)	
Neutral	96 (21%)	62 (21%)	34 (22%)	
Strongly agree	101 (23%)	59 (20%)	42 (27%)	
Strongly disagree	123 (27%)	85 (29%)	38 (24%)	
AI has no limitations in my work				
Agree	57 (13%)	37 (13%)	20 (13%)	0.002
Disagree	71 (16%)	49 (17%)	22 (14%)	
Neutral	90 (20%)	44 (15%)	46 (29%)	
Strongly agree	89 (20%)	65 (22%)	24 (15%)	
Strongly disagree	149 (33%)	106 (36%)	43 (28%)	
Are you in charge of AI in your institution?				
No	45 (10%)	26 (8.9%)	19 (12%)	0.6
Yes	75 (17%)	51 (17%)	24 (15%)	
Someone responsible for AI in education research innovation and integration	367 (82%)	241 (83%)	126 (81%)	0.04
For my work AI is				
A big part of what we do	81 (18%)	51 (17%)	30 (19%)	<0.001
A small part	184 (41%)	98 (34%)	86 (55%)	
I have no idea	40 (8.9%)	20 (6.8%)	20 (13%)	
It is future plan	43 (9.6%)	26 (8.9%)	17 (11%)	
We are not looking at or planning for	107 (24%)	85 (29%)	22 (14%)	
We’re beginner	137 (31%)	88 (30%)	49 (31%)	
Organization have a strategy for AI				
I have no idea	52 (12%)	40 (14%)	12 (7.7%)	
No	69 (15%)	33 (11%)	36 (23%)	<0.001
We’re developing one	175 (39%)	134 (46%)	41 (26%)	
Yes	134 (30%)	96 (33%)	38 (24%)	
What are the biggest challenges for AI implementation in your work?				
Graduates aren’t leaning the AI knowledge and skills at university	72 (16%)	23 (7.9%)	49 (31%)	0.01
It is hard to educate and train the current staff in AI technology	67 (15%)	39 (13%)	28 (18%)	
It is hard to find good education and training courses in AI	76 (17%)	55 (19%)	21 (13%)	
It is hard to implement AI in the work and practice	49 (11%)	37 (13%)	12 (7.7%)	
Knowledge	69 (15%)	48 (16%)	21 (13%)	
The skill development	48 (11%)	36 (12%)	12 (7.7%)	
Which AI relating application do you most need in your work?				
All of the above	101 (23%)	53 (18%)	48 (31%)	
Communication	105 (23%)	63 (22%)	42 (27%)	
Critical care unit (managing critically ill)	3 (0.7%)	3 (1.0%)	0 (0%)	
Dose management (Ex. drug)	34 (7.6%)	22 (7.5%)	12 (7.7%)	0.23
Emergency patient care	81 (18%)	60 (21%)	21 (13%)	
Image interpretation	35 (7.8%)	26 (8.9%)	9 (5.8%)	
Policy and procedures	24 (5.4%)	19 (6.5%)	5 (3.2%)	
Quality control	75 (17%)	51 (17%)	24 (15%)	
Research in respiratory care	1 (0.2%)	1 (0.3%)	0 (0%)	
Teaching and mentoring (respiratory care education)	68 (15%)	39 (13%)	29 (19%)	

Interestingly, academicians demonstrated a higher level of familiarity with AI compared to clinicians. While 44% of academicians reported being familiar with AI, only 19% of clinicians felt the same, though many academicians lacked confidence in applying AI in practice. Additionally, more academicians had attended AI courses (27% vs. 16% of clinicians) and were more aware of the challenges associated with integrating AI into respiratory therapy practices (35% vs. 19% of clinicians). Overall, these findings emphasize the urgent need to incorporate AI education into respiratory care curricula and provide accessible training opportunities. This approach will be vital in preparing the workforce for the growing role of AI in respiratory care.

### Based on the years of experience groups

#### For those with 0–5 years of experience

This group made up 72% of the respondents and seemed generally more familiar with AI concepts (Table 4). However, 18% still had no idea what AI meant. While 22% were excited about integrating AI into respiratory care practice, an equal proportion (22%) remained neutral. Skill development (22%) and knowledge gaps (25%) were seen as the biggest hurdles for AI implementation. The top AI applications needed were research (19%), image interpretation (15%), and critical care management (18%). About 45% reported having someone overseeing AI integration at their institution, but 43% were unaware of any organizational AI strategy.

**Table 4:** Based on the experience about AI in RC domain among the participants.

Characteristic	Overall, *N* = 448	0 to 5, *N* = 327	More than 5, *N* = 121	*p*-value
How well you understood what AI means				
I have no idea	73 (16%)	58 (18%)	15 (12%)	
I’m comfortable with what it means, but I’ve no technical knowledge	124 (28%)	90 (28%)	34 (28%)	
I’m familiar with it but would not confidently apply that knowledge at work	123 (27%)	94 (29%)	29 (24%)	0.036
I’ve read/heard about it in the news, poster or social media	90 (20%)	65 (20%)	25 (21%)	
Very comfortable, I work in AI	38 (8.5%)	20 (6.1%)	18 (15%)	
The RT curriculum should include at least some basic knowledge of AI				
Agree	154 (34%)	111 (34%)	43 (36%)	
Disagree	24 (5.4%)	17 (5.2%)	7 (5.8%)	
Neutral	90 (20%)	72 (22%)	18 (15%)	0.2
Strongly agree	110 (25%)	73 (22%)	37 (31%)	
Strongly disagree	70 (16%)	54 (17%)	16 (13%)	
What is your perception toward integrating AI into RC practice				
Aware of the challenge	110 (25%)	85 (26%)	25 (21%)	0.009
Excited	117 (26%)	71 (22%)	46 (38%)	
I do not know enough	83 (19%)	68 (21%)	15 (12%)	
Neutral	97 (22%)	72 (22%)	25 (21%)	
Worried about the impact	41 (9.2%)	31 (9.5%)	10 (8.3%)	
Knowledge gain				
Attending courses	89 (20%)	66 (20%)	23 (19%)	
No knowledge	63 (14%)	49 (15%)	14 (12%)	
Postgraduate training	54 (12%)	42 (13%)	12 (9.9%)	0.5
Self-taught	151 (34%)	103 (31%)	48 (40%)	
Work-related activities	91 (20%)	67 (20%)	24 (20%)	
AI play an important role in RC				
Agree	121 (27%)	82 (25%)	39 (32%)	
Disagree	29 (6.5%)	23 (7.0%)	6 (5.0%)	
Neutral	129 (29%)	95 (29%)	34 (28%)	0.6
Strongly agree	108 (24%)	80 (24%)	28 (23%)	
Strongly disagree	61 (14%)	47 (14%)	14 (12%)	
AI will take place in many RC applications and practice				
Agree	153 (34%)	112 (34%)	41 (34%)	
Disagree	43 (9.6%)	30 (9.2%)	13 (11%)	
Neutral	94 (21%)	68 (21%)	26 (21%)	0.8
Strongly agree	96 (21%)	68 (21%)	28 (23%)	
Strongly disagree	62 (14%)	49 (15%)	13 (11%)	
AI will threaten/disrupt the respiratory care practice				
Agree	79 (18%)	60 (18%)	19 (16%)	
Disagree	119 (27%)	87 (27%)	32 (26%)	
Neutral	128 (29%)	95 (29%)	33 (27%)	0.6
Strongly agree	47 (10%)	36 (11%)	11 (9.1%)	
Strongly disagree	75 (17%)	49 (15%)	26 (21%)	
AI will threaten/disrupt some respiratory care careers				
Agree	96 (21%)	77 (24%)	19 (16%)	
Disagree	101 (23%)	65 (20%)	36 (30%)	
Neutral	123 (27%)	89 (27%)	34 (28%)	0.08
Strongly agree	57 (13%)	46 (14%)	11 (9.1%)	
Strongly disagree	71 (16%)	50 (15%)	21 (17%)	
AI has no limitations in my work				
Agree	90 (20%)	71 (22%)	19 (16%)	
Disagree	89 (20%)	65 (20%)	24 (20%)	
Neutral	149 (33%)	107 (33%)	42 (35%)	0.6
Strongly agree	45 (10%)	30 (9.2%)	15 (12%)	
Strongly disagree	75 (17%)	54 (17%)	21 (17%)	
Are you in charge of AI in your institution?				
No	367 (82%)	267 (82%)	100 (83%)	0.8
Yes	81 (18%)	60 (18%)	21 (17%)	
Someone responsible for AI in education research innovation and integration	184 (41%)	146 (45%)	38 (31%)	0.011
For my work AI is				
A big part of what we do	40 (8.9%)	29 (8.9%)	11 (9.1%)	
A small part	43 (9.6%)	33 (10%)	10 (8.3%)	
I have no idea	107 (24%)	82 (25%)	25 (21%)	0.2
It is future plan	137 (31%)	98 (30%)	39 (32%)	
We are not looking at or planning for	52 (12%)	31 (9.5%)	21 (17%)	
We’re beginner	69 (15%)	54 (17%)	15 (12%)	
Organization have a strategy for AI				
I have no idea	175 (39%)	142 (43%)	33 (27%)	<0.001
No	134 (30%)	78 (24%)	56 (46%)	
We’re developing one	72 (16%)	56 (17%)	16 (13%)	
Yes	67 (15%)	51 (16%)	16 (13%)	
What are the biggest challenges for AI implementation in your work?				
Graduates aren’t leaning the AI knowledge and skills at university	76 (17%)	60 (18%)	16 (13%)	
It is hard to educate and train the current staff in AI technology	49 (11%)	33 (10%)	16 (13%)	
It is hard to find good education and training courses in AI	69 (15%)	53 (16%)	16 (13%)	0.016
It is hard to implement AI in the work and practice	48 (11%)	27 (8.3%)	21 (17%)	
Knowledge	101 (23%)	82 (25%)	19 (16%)	
The skill development	105 (23%)	72 (22%)	33 (27%)	
Which AI relating application do you most need in your work?				
All of the above	3 (0.7%)	2 (0.6%)	1 (0.8%)	
Communication	34 (7.6%)	26 (8.0%)	8 (6.6%)	
Critical care unit (managing critically ill)	81 (18%)	59 (18%)	22 (18%)	
Dose management (Ex. drug)	35 (7.8%)	32 (9.8%)	3 (2.5%)	
Emergency patient care	24 (5.4%)	22 (6.7%)	2 (1.7%)	0.45
Image interpretation	75 (17%)	50 (15%)	25 (21%)	
Policy and procedures	1 (0.2%)	0 (0%)	1 (0.8%)	
Quality control	68 (15%)	44 (13%)	24 (20%)	
Research in respiratory care	86 (19%)	61 (19%)	25 (21%)	
Teaching and mentoring (respiratory care education)	41 (9.2%)	31 (9.5%)	10 (8.3%)	

#### For those with more than 5 years of experience

This group comprised 27% of respondents and generally exhibited more uncertainty about AI. Only 15% worked directly in AI roles. While 38% were excited about AI integration, 21% were aware of the associated challenges. Skill development (27%) and difficulties in implementation (17%) were flagged as major concerns. The applications with highest AI needs were quality control (20%), image interpretation (21%), and research in respiratory care (21%). Only 31% had someone responsible for AI integration at their workplace, and a significant 46% reported no organizational AI strategy. Overall, the results suggest that while AI integration is viewed positively across experience levels, considerable knowledge gaps and strategic planning deficits exist, especially among more experienced professionals.

## Discussion

Our study found that respiratory therapists (RTs) have varying levels of familiarity and perceptions towards AI. While most RTs acknowledged the importance of AI, there were significant gaps in knowledge and challenges in implementing AI, especially in terms of education, skill development, and organizational strategy. Differences in gender and experience also influenced perspectives, underscoring the need for tailored training programs and strategic planning to effectively incorporate AI into respiratory care practice. This study is one of the first to explore this topic among RTs, and the results indicate that while RTs see the potential benefits of AI, they lack formal training opportunities. Therefore, integrating AI education into the curriculum of respiratory therapy programs is crucial.

The disparities in AI knowledge and utility, based on gender and professional roles are consistent with findings from a study by Castagno and Khalifa (2020), which highlighted a notable disparity in AI comprehension and adoption among healthcare professionals in the UK. Despite the recognized benefits of AI in healthcare, their research revealed limited daily utilization of AI tools like speech recognition (5%) and a majority of respondents (63%) who never incorporated these technologies into their practice, underscoring a gap between the potential of AI and its actual implementation.

Abuzaid et al. (2022) also found a significant knowledge gap in AI among nurses in the United Arab Emirates. Their research showed that more than half (51%) of the participants learned about AI through self-teaching, indicating a lack of formal educational opportunities in this area. Only 20% had attended courses, and a mere 8% had engaged in postgraduate AI education, while 9% admitted to having no knowledge of AI at all. Notably, 75% of the respondents believed that AI education should be integrated into the nursing curriculum, underscoring the perceived importance of formal AI training.

Banerjee et al. (2021) conducted a survey of 210 trainee doctors at UK NHS postgraduate centers in London to evaluate their views on the impact of AI technologies on clinical training and education. The study, conducted between October and December 2020, focused on key training domains such as clinical judgment, practical skills, and research and quality improvement skills. The analysis revealed that a majority (58%) of respondents perceived AI technologies as having a positive overall impact on their training and education. Notably, 62% believed that AI would reduce clinical workload, and 68% felt it would enhance research and audit training. However, there was scepticism about AI’s potential to improve clinical judgment (46% agreement, *p* = 0.12) and practical skills training (32% agreement, *p* < 0.01). A key concern raised was the lack of AI training in current curricula, with 92% of respondents noting this gap, and 81% supporting the inclusion of more formal AI training. While the benefits of AI in healthcare are evident, it is essential to understand the knowledge, attitudes, and practices of respiratory therapists regarding AI implementation in their daily work. Our study identified various factors influencing RTs’ knowledge, perceptions, and practices related to AI use, including gender and age.

Moreover, our research uncovered disparities in knowledge and perceptions regarding the integration of AI between clinicians and academicians, that had not been previously documented scientifically. Academicians demonstrated a greater familiarity with AI compared to clinicians, with a higher proportion of academicians having participated in AI-related courses. Additionally, academicians, who predominantly held master’s or doctoral degrees compared to clinicians (45% vs. 9.6%), were more aware of the challenges in integrating AI into respiratory therapy practice. Similarly, age or years of experience also seemed to impact attitudes toward AI application. Participants with less than 5 years of experience [with the majority being under 25 years old (75.1%)] appeared more familiar with AI concepts compared to participants with more than 5 years of experience, who were relatively older, with 73% aged 31 years and above. This low level of experience was a significant limitation of our study and may have introduced bias, as the perceptions and familiarity with AI of less experienced professionals could differ from those of more seasoned practitioners. This bias arised because the respiratory therapy profession in Saudi Arabia has expanded rapidly over the past 5–7 years, resulting in a predominance of newer professionals. To mitigate this bias in future studies, we recommend broadening recruitment efforts to include a larger proportion of experienced professionals, implementing stratified sampling to ensure diverse representation, and conducting longitudinal studies to track how perceptions evolve with experience.

The top three AI applications sought after by the participating respiratory therapists were in the fields of research (19%), critical care units (18%), and image interpretation (17%). It is worth noting that most participants, regardless of gender, age, or workplace, agreed or strongly agreed that the respiratory therapy curriculum should cover basic AI knowledge. However, a major limitation of the study is the subjective nature of self-reported questionnaires, which may introduce a certain degree of bias in the reported responses. In a related discussion on how AI is perceived among patient populations, a study by Fritsch et al. (2022) found that German patients and their companions are generally open to the use of AI in healthcare. Although their knowledge about AI was moderate, the majority viewed AI in healthcare positively. Importantly, patients emphasized the need for physician supervision over AI, insisting that doctors retain ultimate responsibility for diagnosis and therapy.

## Conclusion

This study found that there are different levels of knowledge and perception about AI, revealing significant challenges such as knowledge gaps and organizational readiness deficits. The use of AI differs between genders and experience levels, suggesting the importance of customized education and training approaches. Addressing these challenges through foundational to comprehensive AI training programs and strategic planning can help improve the integration of AI in respiratory care services may result in enhanced patient outcome. Given that this study is the first of its kind globally, further research is recommended on a broader scale to assess the formal integration and use of AI in respiratory therapy across diverse populations and healthcare systems. Such studies will be crucial in developing universally applicable strategies for AI integration, ensuring that respiratory care professionals worldwide can fully leverage AI’s potential to advance the field.

## Data Availability

The original contributions presented in the study are included in the article/supplementary material, further inquiries can be directed to the corresponding author.
